# The SUMOylation of Human Cytomegalovirus Capsid Assembly Protein Precursor (UL80.5) Affects Its Interaction with Major Capsid Protein (UL86) and Viral Replication

**DOI:** 10.3390/v15040931

**Published:** 2023-04-07

**Authors:** Zhigang Zhang, Sisi Xia, Zhigang Wang, Nina Yin, Jun Chen, Luyao Shao

**Affiliations:** 1 Basic Medical College, Hubei University of Chinese Medicine, Wuhan 430065, China; 2 Department of Biological Engineering, Wuhan Polytechnic University, Wuhan 430023, China; 3 Guangdong Province Key Laboratory of Pharmacodynamic Constituents of TCM and New Drugs Research, College of Pharmacy, Jinan University, Guangzhou 510632, China

**Keywords:** human cytomegalovirus (HCMV), UL80.5, SUMOylation, capsid assembly

## Abstract

Human Cytomegalovirus Capsid Assembly Protein Precursor (pAP, UL80.5) plays a key role in capsid assembly by forming an internal protein scaffold with Major Capsid Protein (MCP, UL86) and other capsid subunits. In this study, we revealed UL80.5 as a novel SUMOylated viral protein. We confirmed that UL80.5 interacted with the SUMO E2 ligase UBC9 (58-93aa) and could be covalently modified by SUMO1/SUMO2/SUMO3 proteins. ^371^Lysine located within a ψKxE consensus motif on UL80.5 carboxy-terminal was the major SUMOylation site. Interestingly, the SUMOylation of UL80.5 restrained its interaction with UL86 but had no effects on translocating UL86 into the nucleus. Furthermore, we showed that the removal of the ^371^lysine SUMOylation site of UL80.5 inhibited viral replication. In conclusion, our data demonstrates that SUMOylation plays an important role in regulating UL80.5 functions and viral replication.

## 1. Introduction

Human cytomegalovirus (HCMV), a member of the herpesvirus family, is a widespread pathogen that affects 70–90% of the general population and can establish lifelong latent infection [1]. Although HCMV infection is generally asymptomatic or mild in immune-competent hosts, it can be life-threatening and cause severe disease complications in immune-compromised hosts [2]. Indeed, HCMV infection is a leading viral cause of congenital abnormalities, intellectual disabilities, and cerebral palsy in newborns [3,4].

HCMV exhibits a characteristic temporal cascade of gene expression with immediate-early (IE), early (E), and late (L) phases [5,6]. UL80.5 as a late-phase protein plays a crucial role in HCMV capsid assembly [7]. Capsid assembly of HCMV is initiated by UL80.5 forming a complex with MCP (UL86) and UL80 in the cytoplasm [8,9]. UL80.5 has two important binding domains, including the CCD domain where UL80.5 interacts with UL86 and the ACD domain where UL80.5 interacts with itself or UL80 [10,11]. By forming a complex, UL80.5 provides the nuclear localization sequences that UL86 lacks and translocates it into the nucleus [12,13]. Once inside the nucleus, UL80.5 further associates with itself and UL80, causing UL86 protomers to coalesce with themselves and other capsid subunits to form a capsid scaffold [14]. This internal scaffold then interacts with the triplex that formed between MnCP (UL85) and MnCP-bp (UL46), leading to the formation of procapsid. Although the scaffold plays a central role in capsid assembly, no scaffolding protein is found within the mature capsid or the virion. Finally, UL80.5 and UL80 are cleaved at the M site and eliminated to make room for viral genome [15,16]. In fact, only a small fraction of the assembled procapsids accomplish the elimination of scaffolding structures and are filled with viral genomic DNA, which becomes infectious C-capsids [17].

SUMOylation is a post-translational protein modification that is analogous to ubiquitination and was discovered in the 1990s [18,19]. There are five SUMO family members (SUMO1, SUMO2, SUMO3, SUMO4, and SUMO5) that have been identified so far, while only SUMO1, SUMO2, and SUMO3 are widely distributed in all human tissue [20]. SUMO2 is 97% similar to SUMO3; therefore, these are commonly classified as SUMO2/3 [21]. In the SUMOylation cascade, SUMO paralogs are expressed as immature propeptides that require cleavage to expose the C-terminal diglycine motif by SUMO-specific proteases (SENPs) [22]. The E1 activating enzyme interacts with SUMO to form a thioester bond in the presence of ATP and then covalently transfers SUMO to E2 ligase UBC9 [23]. Unlike the ubiquitin pathway that contains many E2 enzymes, UBC9 is the sole E2 ligase for SUMO. UBC9 interacts with various substrates and transfers SUMO to lysine residues in target proteins with or without the aid of E3 ligases [24,25]. Typically, the lysine residues subject to SUMOylation are found within a consensus SUMO modification motif ψKxE (usually ψ is a hydrophobic residue, x is any residue, and K is the conjugation site) [26]. In recent years, SUMOylation has been identified as a novel and important posttranslational modification mechanism in a variety of cellular processes, including DNA replication and repair, transcription, nuclear transport, cell–cycle progression, signal transduction, and protein–protein interactions [27,28].

There are multiple mechanisms by which viruses hijack or manipulate cellular the SUMOylation system to accomplish replication. Several viral proteins have been reported as SUMO substrates. For example, HCMV IE1 protein was the first viral protein found to be SUMOylated [29], and SUMOylation-deficient IE1 resulted in an impaired capacity for HCMV replication; the SUMOylation of HPV E2 protein contributed to increased replication by enhancing its stability [30]; and the SUMOylation of ZIKA NS5 protein determined its assembly into discrete nuclear bodies (NBs) to persistently infect host cells [31]. In addition, viruses can manipulate the process of SUMOylation during infection and replication, resulting in changes in cellular SUMOylation levels. Hepatitis C virus (HCV) infection can increase the expression of SUMO1 in the host, which is responsible for HCV replication [32]. The adenoviral E4-ORF3 specifically targets and mislocalizes the SUMO E3 ligases PIAS3 of host cells to facilitate viral replication and infection [33]. Given that SUMOylation has emerged as a key post-translational modification that can be used by viruses to alter viral replication, it could be an ideal drug target for antiviral therapies.

In this study, we reported UL80.5 as a novel SUMOylated herpesviral protein belonging to late-phase proteins. Our findings suggest that the SUMOylation of UL80.5 plays an important role in HCMV assembly and replication and reveal that the SUMOylation of UL80.5 inhibits the interaction between UL80.5 and UL86. These results provide insight into how HCMV hijack cellular pathways to achieve replication within the host.

## 2. Materials and Methods

### 2.1. Cells and Viruses

Human foreskin fibroblasts (HFFs), U251 cells, and HEK293T cells were maintained in Dulbecco’s modified Eagle medium (DMEM) supplemented with 10% fetal bovine serum. HCMV (wild type, mutant, and control Towne-BAC) were generated and propagated in HFFs using a method previously described [34].

### 2.2. Construction of Plasmids

The construct UL80.5/UL86 was derived from the DNA of Towne-BAC [5], while the construct UBC9 and the constructs SUMO1/SUMO2/SUMO3 were extracted from the human fetal brain cDNA library (Clontech, Mountain View, CA, USA). UL80.5 cDNA was sub-cloned into pGBKT7 and pCMV-HA vectors. UBC9 cDNA was sub-cloned into pGADT7, pCMV-Myc, and pRK11-Flag vectors (provided by Dr. Hongbin Shu, Wuhan University, China). Truncated UBC9 mutants were generated by PCR using pGADT7-UBC9 as the template and then inserted into NdeI/BamHI digested pGADT7. SUMO1 cDNA was sub-cloned into pCMV-Myc and pLenti vectors. SUMO2 and SUMO3 cDNA were sub-cloned into pCMV-Myc vectors. The constructs that contained the sequences that encoded different UL80.5 (K-R) mutants were generated by a Fast Mutagenesis kit (Transgen Biotech, Beijing, China) using pCMV-HA-UL80.5 as the template with primers that contained appropriate nucleotide change (Table 1). There were a total of ten lysine residues that individually mutated to arginine which were separately called K41R, K163R, K175R, K178R, K205R, K208R, K242R, K296R, K355R, and K371R. The construct that contained the sequences encoding the UBC9 (C93S) mutant was also generated by a Fast Mutagenesis kit using pRK11-FLAG-UBC9 as the template.

### 2.3. Y2H Analysis

Protein interactions were analyzed using GAL4 fusion proteins in a yeast two-hybrid system. Saccharomyces cerevisiae strain AH109 and control vectors pGADT7, pGADT7-T, pGBKT7-p53, and pGBKT7-Lam were purchased from Clontech. The AH109 yeast strain was transformed with the bait plasmid pGBK-UL80.5 (in fusion with GAL4-BD) and subsequently transformed with selected expression clones pGAD-UBC9 and UBC9 deletion mutant series (in fusion with GAL4-AD). Positive clones were selected on a synthetic dropout medium that lacked four nutrients, including tryptophan, leucine, adenine, and histidine (QDO), and were tested for β-galactosidase activity.

### 2.4. Immunoprecipitation and Immunoblotting

In the SUMO1 denaturing immunoprecipitation assay, cells were prepared in a SDS lysis buffer (50 mM Tris-HCl, pH 7.8, 150 mM NaCl, 1% NP40, 1% SDS, and 10% glycerol) and boiled for 10 min. The lysates were diluted by 10-fold with a Lysis buffer (50 mM Tris-HCl. pH 7.8, 150 mM NaCl, 1% NP40, and 10% glycerol) and an EDTA-free protease inhibitor cocktail (Roche, Basel, Switzerland). Samples were incubated with appropriate antibodies for 3 h or overnight at 4 °C before adding protein G agarose (GE Healthcare, Pittsburgh, PA, USA) for 1 h. After extensive washing, the immunoprecipitates and supernatants were subjected to SDS-PAGE, followed by immunoblotting analysis. In the common immunoprecipitation assay, cells were lysed in a NP40 lysis buffer (50 mM Tris-HCl, pH 7.8, 150 mM NaCl, 0.5% NP40, 0.5% Triton-X100, 5 mM EDTA, and 0.25% deoxycholate) with a cocktail.

The denatured polypeptides were separated by electrophoresis in 4% to 10% polyacrylamide gradient gels which contained SDS (SDS-PAGE) and transferred electrically to nitrocellulose membranes. The detection of tagged proteins was performed using incubation with anti-Myc, anti-HA, or anti-Flag antibodies and IgG antibody conjugated with horseradish peroxidase as the secondary reagent to visualize bound antibodies. The membranes were subsequently stained with a chemiluminescent substrate with the aid of ECL Western blotting detection reagent kits (GE Healthcare, Chicago, IL, USA) and quantitated using a phosphorimager.

### 2.5. Indirect Immunofluorescence

U251 cells that were grown on glass coverslips were cotransfected with plasmids using Lipofectamine 2000 (Invitrogen, Carlsbad, CA, USA). After 48 h of transfection, cells were washed twice with PBS, fixed with 4% paraformaldehyde for 15 min, permeabilized in PBS with 0.1% Triton X-100 for 10 min, blocked in 4% bovine serum albumin (BSA), and then incubated with a mouse anti-flag antibody (1:1000, Merck, Darmstadt, Germany) and a rabbit anti-Myc antibody (1:100, Merck) overnight. Next, the cells were washed with PBS three times and then incubated with a fluorescein (FITC)-tagged donkey anti-rabbit IgG antibody (1:100, Roche) and a rhodamine (TRITC)-tagged goat anti-mouse IgG antibody (1:100, Roche) for 1 h. Nuclear staining was performed with 4, 6-di-amidino-2-phenylindole (DAPI) (Invitrogen). Finally, the cells were observed using a laser scanning confocal microscope.

### 2.6. Lentivirus Production and Infection

We used the 3xFlag sequence to replace the GFP sequence in the pLenti CMV GFP Puro vector (Addgene, 658-5) and added some restriction enzyme cutting sites (XbaI-EcoRV-BstBI-BamHI) before the 3xFlag tag. Then, the pLenti vector encoding the 3xFlag-SUMO1 were transfected into HEK293T cells together with psPAX2 and pMD2.G with a ratio of 4:3:1. Culture supernatants were harvested 36 h and 60 h after transfection. U251 cells were infected with supernatants that contained lentiviral particles in the presence of 4 μg/mL polybrene (Merck). After 48 h of culture, stable transduced cells were selected with 2 μg/mL puromycin (Merck).

### 2.7. BAC Mutagenesis and Recombinant Viruses

The UL80.5 gene (wild type or K371R mutant) was first subcloned from pCMV-HA-UL80.5 (wild type or K371R mutant) to pEM7/Zeo vector (Invitrogen). Next, UL80.5 and Zeo Cassette, which contains the Zeocin resistance marker, were amplified together by PCR. PCR products were used to generate Towne-BAC-UL80.5WT-Zeo and Towne-BAC-UL80.5(K371R)-Zeo by Zeocin selection following a recombination-mediated method that was previously described in [34]. This step introduced a K371R mutation into the viral UL80.5 gene as well as the Zeo cassette right after UL80.5 loci in the strain Towne-BAC. The insertion of K371R and Zeo cassette was confirmed by PCR and sequencing. Original wild type Towne-BAC, Towne-BAC-UL80.5WT-Zeo, and Towne-BAC-UL80.5(K371R)-Zeo were used to generate and propagate three HCMV viruses (WT, UL80.5WT-Zeo, and UL80.5(K371R)-Zeo) in HFFs.

### 2.8. Virus Yield Assay

HFF cells seeded in 6-well plates were infected with HCMV at a multiplicity of infection (MOI) of 0.02 or 0.01 in a 200 μL DMEM inoculum and replaced with DMEM supplemented with 10% fetal bovine serum after 2 h of incubation with cells. To determine the level of viral growth, the cells and medium were harvested at desired days post-infection and stored at −80 °C until all samples were collected. The titers of the viral stocks were determined by plaque assay or quantitative real-time PCR (qPCR).

HFF cells were infected with the appropriate virus (WT, UL80.5(K371R)-Zeo, and UL80.5WT-Zeo) at an MOI of 0.02, and titers of viral stocks were determined by plaque assay. HFF cells in 24-well plates were infected with stock preparations serial 10-fold dilutions (from 10^−1^ to 10^−6^). After 2 h of incubation, warmed DMEM+ 1% agarose were added into these plates. Infected cells were monitored by fluorescence microscopy for the expression of the GFP marker, and the virus titer was calculated as the average number of foci expressing GFP (i.e., plaques) per well (in triplicate) multiplied by the dilution factor (i.e., PFU per ml).

HFF cells were infected with the appropriate virus (UL80.5(K371R)-Zeo and UL80.5WT-Zeo) at an MOI of 0.01, and the total DNA of the viral stocks for examination was extracted using the tissue DNA Kit (Omega, Norcross, GA, USA). The levels of viral DNA (UL83 gene) were then determined by qPCR and normalized to the cellular β-actin gene copies.

## 3. Results

### 3.1. UL80.5 Interacts and Co-Localizes with UBC9

**Figure 1:**
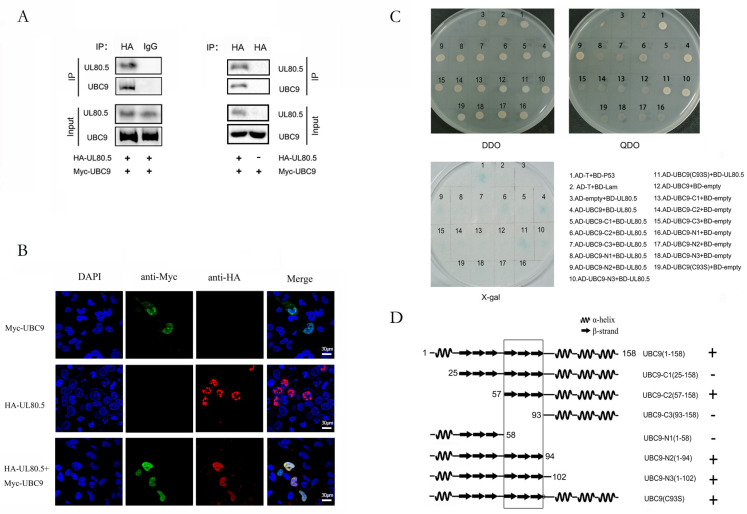
UL80.5 interacts and co-localizes with UBC9. (**A**) HEK293T cells were transfected with Myc-UBC9 with or without HA-UL80.5. Cell lysates were subjected to Co-IP with HA antibody or IgG followed by immunoblot analysis. Data are representative of three independent experiments. (**B**) U251 cells were transfected with HA-UL80.5 alone, Myc-UBC9 alone, or HA-UL80.5 with Myc-UBC9. Subcellular localizations of HA-UL80.5 (red), Myc-UBC9 (green), and the nucleus marker DAPI (blue) were examined by confocal microscopy. The scale bar is 30 μm. Data are representative of three independent experiments. (**C**) Yeast strain AH109 cells were transformed with the combination of BD and AD plasmid, as indicated. Transformed yeast cells were first grown on SD-minus Trp/Leu plates for three days. The colony of yeast was then streaked on SD-minus Trp/Leu/Ade/His plates (QDO) for two days and also subjected to a β-galactosidase activity test(X-gal). BD-p53 and AD-T were used as a positive control and BD-lam and AD-T were used as a negative control. Data are representative of two independent experiments. (**D**) The schematic view and structural features of UBC9 deletion mutants and their interaction with UL80.5 as detected by yeast two-hybrid. +, positive interaction; −, no interaction. The interaction domain was indicated by a box.

To identify the cellular interaction partners of the UL80.5 protein, we performed the yeast two-hybrid system to screen the cDNA library created from human brain using UL80.5 as the bait. After sequencing, one clone that UL80.5 specifically interacted with showed the highest homolog to UBC9, which is an E2 conjugating enzyme in the SUMOylation process. To confirm this interaction in human cells, we performed Co-IP experiments in 293T cells and found that UL80.5 interacted with UBC9 in vivo (Figure 1A). Further, the results of the confocal assay indicated that UL80.5 strongly co-localized with UBC9 in the cell nucleus (Figure 1B).

Though UL80.5 interacted and co-localized with UBC9, whether it got involved in the SUMOylation cascade and what role it played remained unknown. Therefore, we wanted to identify the region of UBC9 where it interacted with UL80.5. The E2 enzyme UBC9 is highly conserved from yeast to human and has a conserved 158-residue αββββββααα motif named as ubc superfold. As shown in Figure 1C, the truncated protein UBC9-C3(1–58) and UBC9-N1(93–158), which lacked β4–β6 regions, did not interact with UL80.5; additionally, the truncated proteins UBC9-C2(57–158), N2(1–94), and N3(1–102), together with full-length UBC9 and the mutant protein UBC9 (C93S), showed interaction with UL80.5 and all possess β4–β6 regions; and the control Y2H experiments showed that none of the truncated UBC9 proteins activated transcription by themselves. The results demonstrated that UBC9 interacted with UL80.5 mainly through the β4–β6 regions (Figure 1D). Former research reported that the interface between UBC9 and the SUMO-E1 is region α1 and the common binding domain for UBC9–E3/substrate interaction is the region below the α1 helix [35]. As the binding domain between UBC9 and UL80.5 is located in the β4–β6 region, we assume that UL80.5 possibly acts as an E3/substrate in SUMOylation cascade.

### 3.2. UL80.5 Is Mainly SUMOylated by SUMO-1 In Vivo

**Figure 2:**
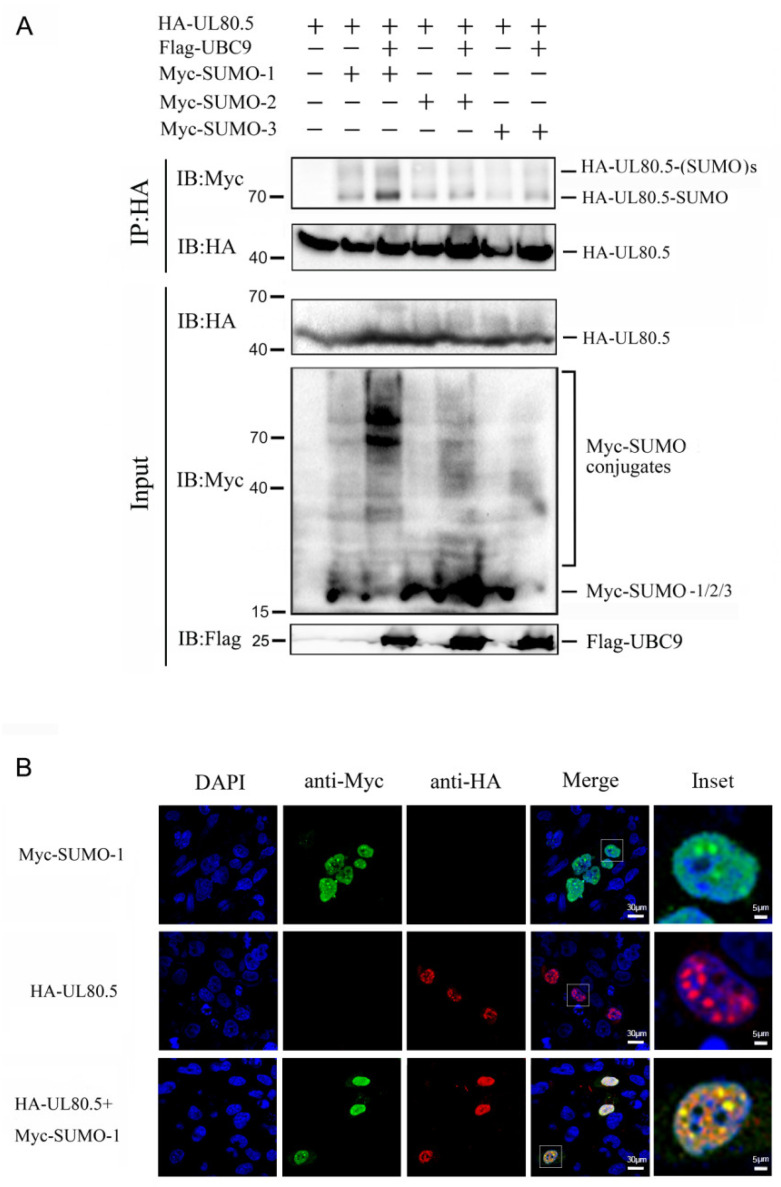
UL80.5 is mainly SUMOylated by SUMO1. (**A**) HEK293T cells were transfected with HA-UL80.5 and Myc-SUMO1/SUMO2 /SUMO3 with or without Flag-UBC9. Cell lysates were subjected to Co-IP with HA antibody followed by immunoblot analysis. Data are representative of three independent experiments. (**B**) U251 cells were transfected with HA-UL80.5 alone, Myc-SUMO1 alone, or HA-UL80.5 with Myc-SUMO1. Subcellular localizations of HA-UL80.5 (red), Myc-SUMO1 (green), and the nucleus marker DAPI (blue) were examined by confocal microscopy. Scale bars for DAPI, anti-Myc, anti-HA, and Merge are 30 μm, and the scale bar for the inset is 5 μm. Data are representative of three independent experiments.

To confirm UL80.5 as a substrate of SUMOylation in mammalian cells and determine which sumo protein or proteins it covalently bonds, a Co-IP assay was performed. The results revealed that the bands which corresponded to SUMO1/2/3-conjugated UL80.5 (70 kDa and 90 kDa) were immunoprecipitated and detected in the presence of SUMO1/2/3 (Figure 2A, lanes 2, 4, 6) and further promoted in the presence of SUMO1/2/3 and UBC9 (Figure 2A, lanes 3, 5, 7), which indicates that UL80.5 could be SUMOylated by SUMO1/2/3 proteins. Moreover, SUMO2/3-conjugated UL80.5 (Figure 2A, lanes 4–7) were detected much less than SUMO-1-conjugated UL80.5 (Figure 2A, lanes 2 and 3). These data suggested that UL80.5 was SUMOylated by SUMO1/SUMO2/SUMO3 via UBC9 in 293T cells and the SUMO-1 modification was the strongest. Furthermore, the results of the confocal assay indicated that UL80.5 strongly co-localized with SUMO1 in the cell nucleus (Figure 2B). Notably, UL80.5 and SUMO1 were mainly distributed as spots in the cell nucleus, and the co-localization also mainly existed in those spots.

### 3.3. SUMOylation Site of UL80.5 Is K371

**Figure 3:**
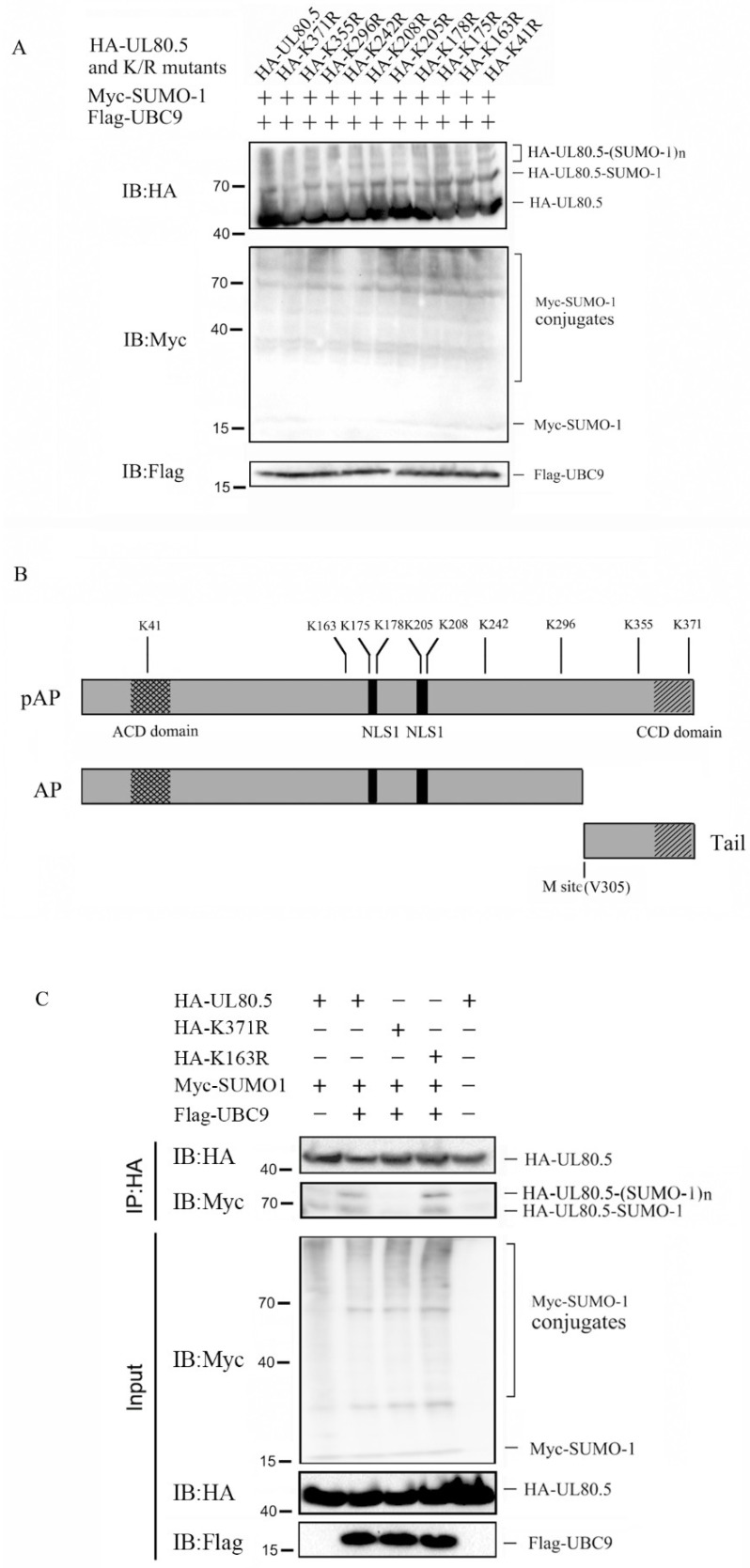
Identification of SUMOylation site of UL80.5. (**A**) HEK293T cells were transfected with Myc-SUMO1 and Flag-UBC9, along with HA-UL80.5 or its HA tagged lysine mutants. Cell lysates were analyzed by immunoblot analysis using corresponding antibodies, as indicated. Data are representative of four independent experiments. (**B**) A diagram of the full-length UL80.5 protein and cleaved forms (AP and tail proteins) is reported, and important functional domains are indicated. Numbers refer to the 10 lysine residues of UL80.5. (**C**) HEK293T cells were transfected with HA-UL80.5, HA-K371R, or HA-163R, with or without Myc-SUMO1 and Flag-UBC9. Cell lysates were subjected to Co-IP with HA antibody, followed by immunoblot analysis. Data are representative of three independent experiments.

Next, we sought to identify the SUMO1 acceptor sites of UL80.5. Since SUMOylation often occurs at the lysine residue of substrates containing ΨKxE/D motif, a prediction analysis by the SUMOsp 2.0-SUMOylation Site Prediction program (http://SUMOsp.biocuckoo.org/index.php, accessed on 1 January 2023) was used and identified two putative SUMOylation sites: K371 and K242. However, the SUMOylation sites also had a chance to be other lysine residues as former reports on the HCMV UL44 protein [34,36]. To test whether one or more of these lysine residues could be SUMO1 acceptor sites, each of the 10 lysine residues was conservatively and individually mutated to arginine. These lysine residues were located in several important domains of UL80.5 (Figure 3B), such as the ACD/CCD domains and the nuclear localization signals (NLS1 and NLS2). Then, lysine mutants were individually tested to determine whether they could be modified by SUMO1 in 293T cells. The results showed that the K371R mutant blocked the SUMOylation of UL80.5 as the band corresponding to SUMOylated UL80.5 (70 kDa, 90 kDa, and 110 kDa) was not detected (Figure 3A, lane 2), while other K/R substitutions showed modification patterns identical to wild type UL80.5 (Figure 3A, lane 1 and lanes 3–11). To further confirm that SUMOylation was responsible for the observed migrating change of UL80.5, we compared the differences in HEK293T cells that were transfected with HA-UL80.5/K371R/K163R in the presence or absence of UBC9 or SUMO1, and a Co-IP assay was performed with HA antibody. With the absence of SUMO1 and UBC9, no SUMO1 conjugated-UL80.5 was immunoprecipitated and detected (Figure 3C, lane 5); with SUMO1 present, SUMO1 conjugated-UL80.5 was immunoprecipitated (Figure 3C, lane 1); with SUMO1 and UBC9 present, more SUMO1 conjugated-UL80.5 was immunoprecipitated (Figure 3C, lane 2); with SUMO1 and UBC9 present, the same amount of SUMO1 conjugated-K163R was immunoprecipitated (Figure 3C, lane 4); and nearly no SUMO1 conjugated-K371R was immunoprecipitated (Figure 3C, lane 3). These results suggested that UL80.5 possesses lysine residue K371 as a major SUMO1 acceptor site.

### 3.4. SUMOylation of UL80.5 Restrains Its Interaction with UL86

**Figure 4:**
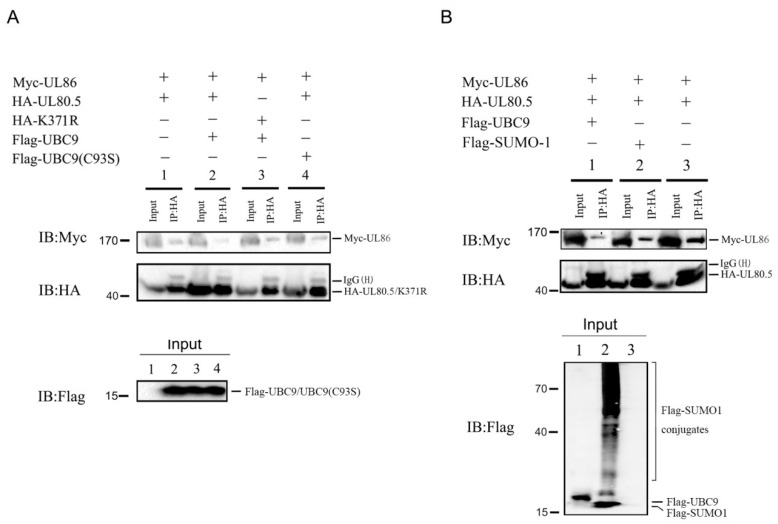
SUMOylation of UL80.5 restrains its interaction with UL86. (**A**) HEK293T cells were transfected with Myc-UL86, HA-UL80.5, or HA-K371R with or without Flag-UBC9 or Flag-UBC9(C93S). Cell lysates were subjected to Co-IP with HA antibody followed by immunoblot analysis. Data are representative of four independent experiments. (**B**) HEK293T cells were transfected with Myc-UL86 and HA-UL80.5 with or without Flag-UBC9 or Flag-SUMO1. Cell lysates were subjected to Co-IP with HA antibody, followed by immunoblot analysis. Data are representative of three independent experiments.

Since the SUMOylation site of UL80.5 had lysine residue K371 in the CCD domain (UL86 binding domain), we wanted to confirm whether this SUMOylation affected its interaction with UL86 (MCP). Competitive Co-IP experiments in 293T cell were performed by co-transfection of HA-UL80.5/K371R and Myc-UL86 in the presence or absence of UBC9 or UBC9(C93S). UBC9(C93S) was the mutant that E2 enzyme activity abolished. The results showed that UL86 was co-precipitated with UL80.5 (Figure 4A, lane 1); the amount of co-precipitated UL86 decreased with the overexpression of UBC9 (Figure 4A, lane 2 vs. lane 1); the amount of co-precipitated UL86 stayed the same with the overexpression of UBC9(C93S) (Figure 4A, lane 4 vs. lane 1); and K371R mutant blocked the SUMOylation of UL80.5 and restored the amount of co-precipitated UL86 (Figure 4A, lane 3 vs. lane 2). Next, we explored whether SUMO1 as the SUMOylation system enhancer affected the interaction of UL80.5 with UL86. The results of the Co-IP assay in 293T cells demonstrated that SUMO1 overexpression inhibited the function of UL80.5 binding UL86 (Figure 4B, lane 2 vs. lane 3), which was the same as UBC9 overexpression (Figure 4B, lane 1 vs. lane 3). In addition, UBC9 had stronger inhibition than SUMO1 as the amount of co-precipitated UL86 was much less. In conclusion, these results demonstrated that SUMOylation of UL80.5 restrains its interaction with UL86.

### 3.5. SUMOylation of UL80.5 Had No Effect on Translocating UL86 into Cell Nucleus

**Figure 5:**
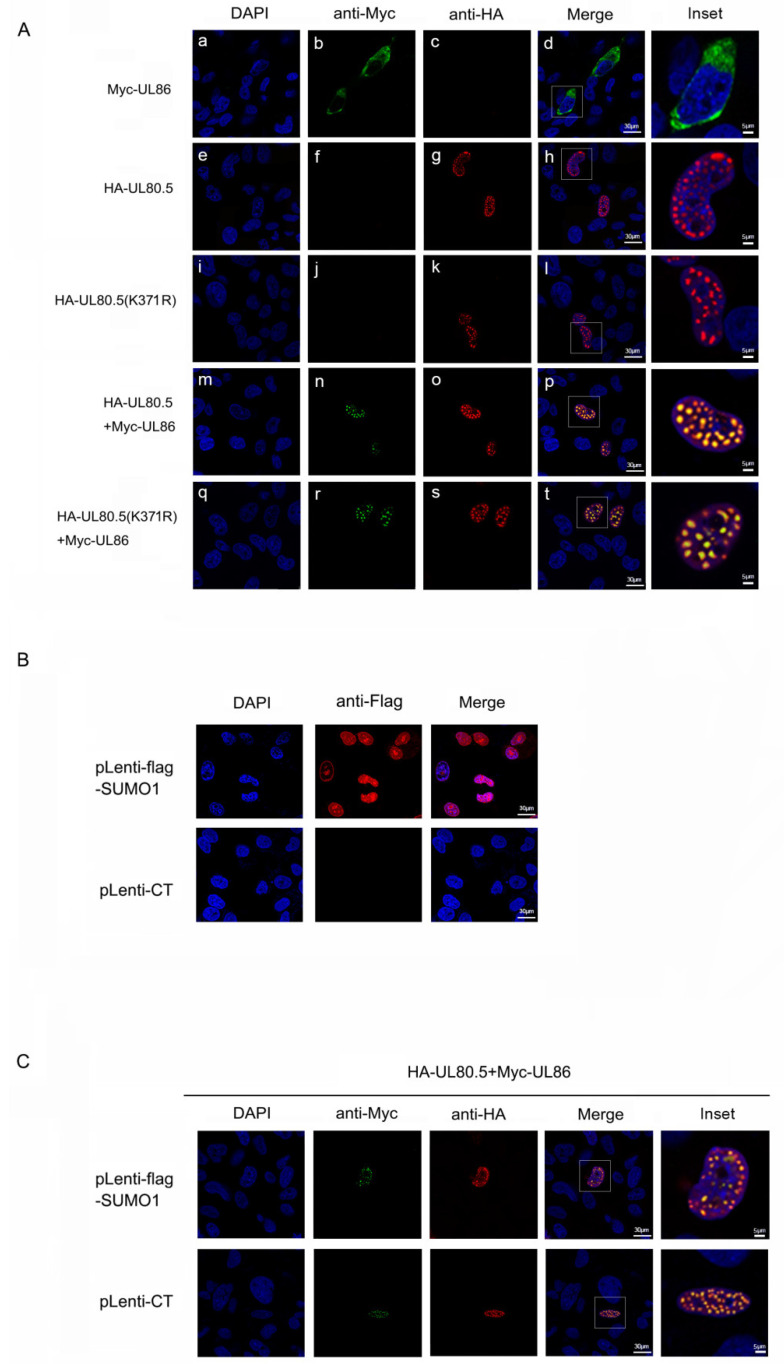
SUMOylation of UL80.5 has no effect on translocating UL86 into cell nucleus. (**A**) U251 cells were transfected with HA-UL80.5/HA-K371R alone, Myc-UL86 alone, or HA-UL80.5/HA-K371R and Myc-UL86. Subcellular localizations of HA-UL80.5/HA-K371R (red), Myc-UL86 (green), and the nucleus marker DAPI (blue) were examined by confocal microscopy. Scale bars for DAPI, anti-Myc, anti-HA, and Merge are 30 μm, and the scale bar for inset is 5 μm. Data are representative of three independent experiments. (**B**) U251 cells were infected with lentivirus expressing flag-SUMO1 (pLenti-flag-SUMO1) or pLenti-CT and selected with puromycin; thus, SUMO1 was stably overexpressed or not. Subcellular localizations of flag-SUMO1 (red) and the nucleus marker DAPI (blue) were examined by confocal microscopy. The scale bar is 30 μm. Data are representative of two independent experiments. (**C**) pLenti-flag-SUMO1 and pLenti-CT stably U251 cells were transfected with HA-UL80.5 and Myc-UL86. Subcellular localizations of HA-UL80.5 (red), Myc-UL86 (green), and the nucleus marker DAPI (blue) were examined by confocal microscopy. Scale bars for DAPI, anti-Myc, anti-HA, and Merge are 30 μm, and the scale bar for inset is 5 μm. Data are representative of three independent experiments.

UL80.5 interacts with UL86 and provides NLS that UL86 lacks to translate it into cell nucleus in the initial assembly process [12]. Since SUMOylation of UL80.5 inhibits the interaction between UL80.5 and UL86, we wanted to determine whether SUMOylation affected the ability of UL80.5 translocating UL86. The data from the confocal assay showed that when UL80.5 or K371R was expressed individually, it was mainly distributed as spots in the cell nucleus (Figure 5A(e–l)); when UL86 was expressed individually, it was distributed in the cytoplasm (Figure 5A(a–d)); when UL80.5 was co-expressed with UL86, UL86 was translocated into the cell nucleus, and the strong co-localization as spots was observed (Figure 5A(m–p)); and the SUMOylation site mutant K371R blocked its SUMOylation, but the same strong co-localization as spots was also observed (Figure 5A(q–t)). Furthermore, the role of SUMO1 as an enhancer of UL80.5 SUMOylation was evaluated in stable cell lines in which U251 cells were infected with lentivirus expressing flag-SUMO1 (Lentivirus-flag-SUMO1) and selected with puromycin. The indirect immunofluorescence assay data showed that pLenti-flag-SUMO1 cells stably expressed flag-SUMO1 (Figure 5B). UL80.5 and UL86 were co-expressed in pLenti-flag-SUMO1 or pLenti-CT U251 cells, and the same strong co-localization as spots of UL80.5 and UL86 was observed in confocal assay (Figure 5C). These experiments demonstrated that the SUMOylation of UL80.5 did not affect its location in the cell nucleus and the ability of translocating UL86 into the nucleus.

### 3.6. Mutation of UL80.5 ^371^lysine Attenuated Progeny Production of HCMV Infection in HFFs

**Figure 6:**
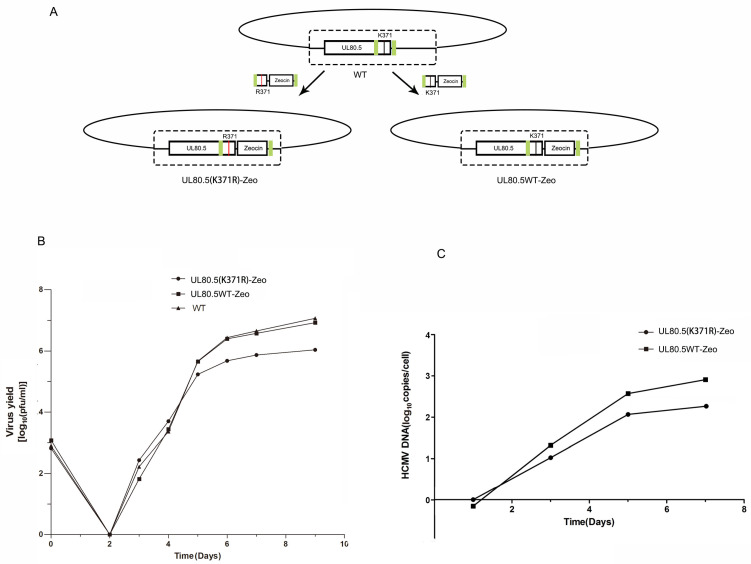
Mutation of ^371^lysine attenuates HCMV replication in HFFs. (**A**) Schematic for BAC construction and generation of wild type, mutant, and control HCMV virus. (**B**) The replication and spread abilities of HCMV were determined by multi-step virus growth curves. HFF cells were infected with an appropriate virus (WT, UL80.5(K371R)-Zeo, and UL80.5WT-Zeo) at a MOI of 0.02 and collected at the indicated time points. The released virus at each time point was titrated on HFFs by serial dilutions and plaques counting to generate growth curves. Data are representative of three independent experiments. (**C**) HFF cells were infected with an appropriate virus (UL80.5(K371R)-Zeo, and UL80.5WT-Zeo) at a MOI of 0.01. Viral DNA synthesis was determined by quantitative real-time PCR at each of the time points indicated. The amount of viral DNA assayed is represented as copies of the viral gene UL83 per copy of the cellular β-action gene.

We further examined whether the SUMOylation of UL80.5 played a role in viral replication. Since other HCMV proteins (e.g., IE1 and IE2) can be SUMOylated and influence viral replication, instead of over-expressing SUMO1, we used mutant viruses which allowed us to directly dissect the SUMO effects on UL80.5. To make an HCMV virus containing UL80.5-K371R single-site mutation, Zeocin cassette was introduced following the UL80.5 loci on the Towne-BAC for the recombination selection purpose (Figure 6A). Multi-step viral growth curve experiments were performed using wild type (WT), UL80.5(K371R)-Zeo mutant and UL80.5WT-Zeo viruses in HFF cells and examined by plaque assay. The inoculum titers are presented in Figure 6B as virus yields on day zero and show that similar amounts of the virus were used. The UL80.5(K371R)-Zeo mutant showed about 2-fold-decreased virus yields at 5 dpi compared to those of wt or UL80.5WT-Zeo viruses, and the trend extended to about 10-fold at 9 dpi (Figure 6B). A similar trend was found in viral replication examined by QPCR for viral DNA synthesis as the UL80.5(K371R)-Zeo mutant showed a decreased amount of viral DNA compared with the UL80.5WT-Zeo virus (Figure 6C). The amount of viral DNA was represented as copies of the viral gene UL83 per copy of the cellular β-action gene. Together, these results suggested that the removal of ^371^lysine SUMOylation site of UL80.5 inhibited viral production. Consequently, the SUMOylation of UL80.5 had a positive effect on HCMV replication.

## 4. Discussion

UL80.5 is an essential gene of HCMV and plays a key role in viral capsid assembly [7,37]. To better understand the biological function of UL80.5, we performed a yeast two-hybrid screen, and the most frequently isolated UL80.5-interacting protein was the SUMO conjugating enzyme UBC9. In this study, we first reported that UL80.5 is a novel target of SUMOylation and could be effectively conjugated with SUMO1, SUMO2, and SUMO3. The conjugation levels of UL80.5 by SUMO1, SUMO2, and SUMO3 proteins were not the same as SUMO1 modification was the strongest. Moreover, several additional higher molecular weight UL80.5 species were detected, suggesting that UL80.5 could be multi-SUMOylated. In mammalian cells, some substrates of SUMOylation were capable of being modified by either SUMO1 or SUMO2/3, and other substrates showed a clear preference for a particular SUMO type [38,39]. Until recently, there were no obvious differences in protein functional types that were modified by SUMO1 versus SUMO2/3. Therefore, we choose SUMO1 modification as a model for our research.

Full length UL80.5 contains 10 lysine residues. A prediction sequence analysis identified the most putative SUMOylation sites, including K371 (middle-possibility) and K242 (low-possibility). We confirmed that the major SUMOylation site of UL80.5 is K371 in the CCD domain. The CCD domain is highly conserved and essential for the interaction between UL80.5 and UL86. Our study demonstrates that the SUMOylation of UL80.5 inhibited its interaction with UL86. The interaction between UL80.5 and UL86 is essential for the process of capsid assembly and formation. First, UL86 needs to be translocated into the nucleus by its interaction with UL80.5; next, the formation of the procapsid shell needs the interaction between UL86 and UL80.5 to coalesce capsid subunits; finally, UL80.5 and UL80 need to cleave themselves through the M site and break their interaction with UL86 to make room for viral DNA. Confocal assay results demonstrated that a lack of SUMOylation of UL80.5 also translocated UL86 into the cell nucleus, which indicated that the SUMOylation did not affect the first step. In addition, we also found UL80.5 co-localized with SUMO1 as spots in the nucleus. It is possible that the SUMO modification of UL80.5 mainly occurred in the nucleus and created no effect on translocating UL86 from the cell cytoplasm into the nucleus.

Past research about SUMOylated viral structural proteins have reported that SUMOylation could have negative or positive impacts on viral assembly and reproduction. For example, the SUMOylation of HIV p6 protein has a negative impact and correlates with reduced viral reproduction through an unknown mechanism [40]. However, for the M1 protein of the influenza A virus (IAV), the SUMOylation of M1 plays a critical role and facilitates viral assembly, and viruses carrying SUMO-deficient M1 produce a lower viral titer [41]. There are two possible theories of positive or negative impacts that exist for the SUMOylation of UL80.5. To confirm whether the SUMOylation of UL80.5 has positive or negative impact, we performed viral growth curve experiments and found out that the SUMOylation site mutant virus (K371R mutant virus) showed restrained viral replication. We hypothesized that the SUMOylation of UL80.5 inhibited the interaction between UL80.5 and UL86, which may promote the removal of UL80.5 from the procapsid to make room for viral DNA (the final step), leading to the completion of capsid assembly and viral multiplication. Nevertheless, there is no direct evidence regarding how the SUMOylation of UL80.5 affected capsid assembly; therefore, this assumption needs more evidence based on future studies.

A previous study showed that the overexpression of SUMO1 enhances virus production in HCMV infected cells [42], which is consistent with our results. Moreover, many herpesvirus proteins are authentic substrates for SUMOylation. IE1 (or IE72), the first and most abundantly expressed IE protein, was the first viral protein found to be SUMOylated [29]. IE2 (or IE86), which is also an IE protein, was later reported to be SUMOylated [43]. The SUMOylation of IE1 contributes to efficient HCMV replication by promoting the accumulation of IE2 [44]. Regarding IE2, research reported that SUMOylation-negative IE2 strongly impaired replication [45]. Furthermore, UL44 as HCMV DNA polymerase subunit was SUMOylated, and its SUMOylation attenuates DNA replication [34]. In conclusion, SUMOylated HCMV proteins have been reported to be involved in transcriptional regulation (IE phase) and DNA replication (E phase) but never capsid assembly (L phase). In this study, we first identified UL80.5 as a late-phase viral protein that can be SUMOylated, which implies that SUMO modification is involved throughout the entire process of HCMV infection. Together, these results may indicate that SUMOylation has great effects on HCMV protein function and overall viral replication or HCMV may use the cellular SUMOylation system to facilitate and complete viral replication.

In this study, we found that UL80.5 is mainly SUMOylated by SUMO1, and the SUMOylation site is K371. This SUMOylation leads to its weaker interaction with MCP (UL86), which is essential for capsid assembly and maturation; however, it does not affect its translocation of UL86 into the nucleus. Furthermore, the UL80.5 SUMOylation site mutant virus shows restrained viral replication, revealing that the SUMOylation of UL80.5 is necessary or has a positive effect on HCMV replication. These results provide insight into how HCMV utilize cellular pathways to achieve optimal production within the host. However, how the SUMOyaltion of UL80.5 directly influences HCMV capsid assembly remains unknown.

## Figures and Tables

**Table 1:** Primers used to individually mutate all ten lysine residues of UL80.5 to arginine.

Position	Primer F Sequence (5′ to 3′)	Primer R Sequence (5′ to 3′)
K41	GAGTTTATTTACCCAGAGACGCTTTT	CTGGGTAAATAAACTCCGTCGTGAGG
K163	GTGGTCAGTCGCAGAGGCAGCACCGT	CTCTGCGACTGACCACGGTGACTACC
K175	GCGGCGGACACAACAGACGCCGTAAG	CTGTTGTGTCCGCCGCTGCCCCCGTG
K178	ACAACAAACGCCGTAGGGAAGCCGCG	CTACGGCGTTTGTTGTGTCCGCCGCT
K205	ACGGCCGGGCGCGAAGGCGTCTAAAA	CTTCGCGCCCGGCCGTGCTCGGCCTC
K208	CGCGAAAGCGTCTAAGAAGTCACGTC	CTTAGACGCTTTCGCGCCCGGCCGTG
K242	CCATTCACGAGCTGAGACGCGATCTG	CTCAGCTCGTGAATGGCATCCCGCAG
K296	TATCAGGAGGTGCCAGGGTAGCTGAG	CTGGCACCTCCTGATAGAAGTGCCGT
K355	CCCAGAGCCCGCCCAGAGACATGGTG	CTGGGCGGGCTCTGGGAAGCTGCGGC
K371	TGGCTGCGCTCAATAGGCTCGAGTAA	CTATTGAGCGCAGCCACAAAAATCCG

## Data Availability

The data presented in this study are available on request from the corresponding author.
